# Primary malignant orbit melanoma

**DOI:** 10.1016/S1808-8694(15)31033-8

**Published:** 2015-10-19

**Authors:** Lucas Gomes Patrocínio, Clauber Lourenço, Cristiane do Prado Silva, Daniela Borges Barra, José Antônio Patrocínio

**Affiliations:** a MD. Otorhinolaryngology Resident - Federal University of Uberlândia.; b Medical Student - Medical School - Federal University of Uberlândia.; c Medical Student - Medical School - Federal University of Uberlândia.; d Medical Student - Medical School - Federal University of Uberlândia.; e Full Professor of Otorhinolaryngology - Medical School - Federal University of Uberlândia. Department of Otorhinolaryngology - Medical School - Federal University of Uberlândia, Minas Gerais

**Keywords:** orbit exenteration, melanoma, eye neoplasia, orbit

## INTRODUCTION

The first case of extracutaneous melanoma was described in Germany (1856) and up until 2001 approximately 1000 cases had been published^1^, ^2^. These are rare lesions, representing about 0.09% of the extracutaneous malignant neoplasias^1^. In the orbit, it is frequently secondary to invasions by conjunctiva, choroid melanomas or from adjacent regions, or blood-born metastases^3^. The primary orbital melanoma represents less than 1% of the primary orbital neoplasias^4^.

## CASE REPORT

O.S.S., female, 64 years old, presented progressive ptosis, visual blurring and right eye scotomatas for two months. Did not present with any otorhinolaryngological complaint. She had proptosis, visual acuity for hands movement, papilledema and extrinsic muscle paralysis to the right side; without alterations noticed at the nasofibroscopy.

**Figure 1:**
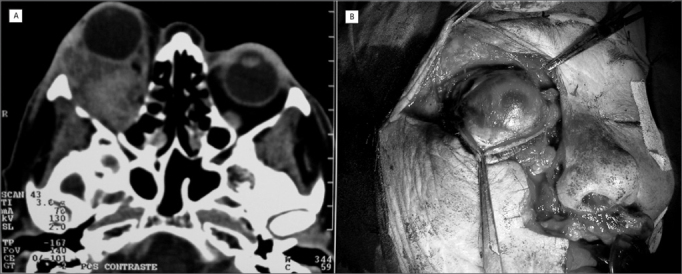
Photography showing an orbit primary melanoma in a CT Scan axial view (A) and during exenteration of the right orbit via broadened Weber-Fergusson incision towards the eyebrow region (B).

CT scan showed a right side retroorbital tumor (Figure 1ª); transconjunctival biopsy (inferior fornix) showed malignant melanoma. Skull, chest and abdomen CT scan did not find other involvements.

Right side exenteration was carried out through a Weber-Fergusson incision broadened towards the eyebrow region (Figure 1b). Histopathology proved that there was no invasion of eye tissue or the optic nerve, without invasion of adjacent structures and free margins.

Four weeks later, radiotherapy started, with 30 applications of 180 cGy. The patient has been followed for 18 months now, with periodic CT scans used for the early identification of metastases or local recurrence.

## DISCUSSION

Extracutaneous melanomas are neoplasias that affect the elderly. Series of primary orbital melanomas show ages varying between 12 and 84 years^3^, ^5^. There are only two cases of African-descendant patients^3^, ^4^, ^5^.

Orbital primary melanomas are probably originated from the congenital remains of cells from the neural crest, and may be found along ciliary nerve, scleral emissary veins or the leptomeninx of the optical nerve^3^, ^4^, ^5^, ^6^. Due to the small number of cases, there is not much data regarding its clinical behavior, however, the most common clinical presentation is pain-associated proptosis originated from a diffuse orbital mass^4^. For diagnostic confirmation it is necessary to have biopsy and immunephenotyping^1^.

In order to define whether the orbital melanoma is primary, it is necessary to show, though image and pathology exams, that it did not originate from the eye globe and it does not represent a metastasis^3^, ^5^, ^6^. Differential diagnosis must be made with benign and malignant tumors of the nose, the paranasal cavities, orbit, and skull base, specially vascular anomalies and pigmentary schwannoma^5^.

Treatment of choice is based on exenteration, that is, complete removal of the orbital content, including the eyeball and eyelids. Radiotherapy and chemotherapy have been used as additional treatment, with uncertain results.^3^, ^4^, ^5^.

## FINAL COMMENTS

The orbit primary melanoma is a tumor of rare occurrence; however it is most of the times fatal. We stress the need to carry out strict clinical and image exams, and specially biopsy followed by immunephenotyping when facing a case of subacute proptosis.
